# Supplementary material for: Natalizumab Induces Changes of Cerebrospinal Fluid Measures in Multiple Sclerosis
Source: Diagnostics (Basel). 2021 Nov 29;11(12):2230. doi: 10.3390/diagnostics11122230 (PMC8699923; doi:10.3390/diagnostics11122230)
Supplement: Supplementary file 1 [file diagnostics-11-02230-s001.zip › diagnostics-1458374-supplementary.pdf]

Supplementary Table 1. Spearman´s correlation between demographic, clinical and MRI measures and CSF characteristics at baseline.

CSF characteristics at baseline	Age	Disease duration	EDSS	T2-LV (ml) (n=64)	BPF (%) (n=64)
CSF-leukocytes (in mcL)	**-0.35****	**-0.44*****	**-0.41*****	-0.14	**0.52*****
CSF-total protein (mg/l)	0.06	-0.13	-0.10	-0.13	0.21
Albumin quotient	0.08	-0.09	-0.06	-0.12	0.1
CSF-restricted total OCB	-0.03	-0.06	-0.08	-0.10	0.18
IgG index	-0.14	-0.15	-0.14	0.13	0.11
IgM index (n=88)	-0.25	0.002	-0.14	0.01	0.06

Legend: BPF = brain parenchymal fraction; CSF = cerebrospinal fluid; EDSS = expanded disability status scale; IgG = immunoglobulin G; IgM = immunoglobulin M; mcL = microliter; OCB = number of oligoclonal bands; T2-LV = T2 lesion volume.

Values entered indicate correlation coefficient. In bold are p-values <0.05 after Benjamini-Hochberg correction for false discovery rate [number of comparisons was 5].

*<0.05

**<0.01

***<0.001

Supplementary Table 2. Predictors of CSF changes over time in multivariable models adjusted also for baseline levels of CSF-measures.

Dependent variable	Independent variables	Statistical results* β (95% CI); p-value
CSF leukocytes at timepoint (mcL)†	Natalizumab treatment	-0.76 (-0.88, -0.64); **<0.0001**
	CSF leukocytes at baseline†	0.56 (0.46, 0.65); **<0.0001**
	Age at baseline CSF exam	0.00 (-0.01, 0.01); 0.99
	Disease duration at baseline CSF exam†	0.03 (-0.07, 0.12); 0.57
	Years between baseline and follow-up CSF exams	0.01 (-0.03, 0.04); 0.65
	EDSS†	0.00 (-0.24, 0.25); 0.98
Albumin qoutient at timepoint	Natalizumab treatment	-0.43 (-0.70, -0.16); **0.002**
	Albumin qoutient at baseline	0.75 (0.67, 0.83); **<0.0001**
	Age at baseline CSF exam	0.02 (0.00, 0.04); **0.032**
	Disease duration at baseline CSF exam†	0.00 (-0.21, 0.21); 0.99
	Years between baseline and follow-up CSF exams	0.13 (0.04, 0.21); **0.004**
	EDSS†	-0.03 (-0.55, 0.50); 0.92
CSF-restricted total OCB at timepoint	Natalizumab treatment	-2.02 (-3.09, -0.95); **0.0003**
	CSF-restricted total OCB at baseline	0.80 (0.69, 0.90); **<0.0001**
	Age at baseline CSF exam	-0.01 (-0.08, 0.06); 0.74
	Disease duration at baseline CSF exam†	0.04 (-0.75, 0.83); 0.91
	Years between baseline and follow-up CSF exams	-0.31 (-0.62, 0.00); 0.053
	EDSS†	1.04 (-1.02, 3.09); 0.33
IgG index at timepoint	Natalizumab treatment	-0.24 (-0.34, -0.15); **<0.0001**
	IgG index at baseline	0.61 (0.54, 0.68); **<0.0001**
	Age at baseline CSF exam	0.00 (-0.01, 0.01); 0.96
	Disease duration at baseline CSF exam†	0.03 (-0.05, 0.10); 0.51
	Years between baseline and follow-up CSF exams	-0.01 (-0.05, 0.02); 0.51
	EDSS†	-0.02 (-0.21, 0.17); 0.85
IgM index at timepoint†	Natalizumab treatment	-0.04 (-0.06, -0.02); **0.0001**
	IgM index at baseline	0.82 (0.75, 0.87); **<0.0001**
	Age at baseline CSF exam	0.00 (0.00, 0.00); 0.60
	Disease duration at baseline CSF exam†	0.00 (-0.02, 0.01); 0.85
	Years between baseline and follow-up CSF exams	0.00 (-0.01, 0.00); 0.71
	EDSS†	0.04 (0.00, 0.08); **0.031**

Legend: β = estimate of linear mixed model; CI = confidence interval; CSF = cerebrospinal fluid; EDSS = expanded disability status scale; IgG = immunoglobulin G; IgM = immunoglobulin M; OCB = number of oligoclonal bands; QAlb = albumin quotient; mcL = microliter.

* In bold are p-values <0.05 (p-values from the multivariate models investigating independent associations between predictors and MRI outcomes were not corrected for FDR). Associations were tested by using the linear mixed model analysis with a fixed slope and random intercept specified for each patient.

† log+1 transformed variable

Supplementary Table 3. Predictors of annualized brain volume loss and T2 lesion volume change over follow-up.

Dependent variable	Independent variables	Statistical results β (95% CI); p-value
Annualized whole brain % volume change*	CSF leukocytes (mcL) † (n=64)	-0.31 (-0.61, -0.02); 0.034
	Albumin qoutient (n=64)	0.07 (-0.74, -0.11); 0.20
	CSF-restricted total OCB (n=64)	-0.02 (-0.06, 0.02); 0.32
	IgG index † (n=61)	-0.32 (-0.72, 0.08); 0.10
	IgM index † (n=62)	-0.46 (-0.74, -0.18); **0.001**
Annualized T2 lesion** volume (ml) volume change	CSF leukocytes (mcL) † (n=45)	0.00 (-0.61, 0.60); 0.99
	Albumin qoutient (n=45)	-0.01 (-0.22, 0.21); 0.95
	CSF-restricted total OCB (n=45)	-0.02 (-0.11, 0.08); 0.72
	IgG index † (n=45)	-0.20 (-1.01 , 0.61); 0.62
	IgM index † (n=40)	0.08 (-0.43, 0.60); 0.74

Legend: β = estimate of linear mixed model; CI = confidence interval; CSF = cerebrospinal fluid; IgG = immunoglobulin G; IgM = immunoglobulin M; OCB = number of oligoclonal bands; mcL = microliter.

*In bold are p-values <0.05 after correction by Benjamini-Hochberg procedure. Associations were tested by using the linear mixed model analysis with a fixed slope and random intercept specified for each patient adjusted age, disease duration † and expanded disability status scale (EDSS) † at baseline and years between baseline and follow-up timepoint.

† log [x+1] transformed variable

* Annualized whole brain volume change (in %, analyzed by SIENA) over follow-up (n=64)
** Annualized T2 lesion volume absolute change (in ml; analyzed by ScanView) over follow-up (n=45)
